# Effects of silver nanoparticles and ions on a co-culture model for the gastrointestinal epithelium

**DOI:** 10.1186/s12989-016-0117-9

**Published:** 2016-02-17

**Authors:** Anastasia Georgantzopoulou, Tommaso Serchi, Sébastien Cambier, Céline C. Leclercq, Jenny Renaut, Jia Shao, Marcin Kruszewski, Esther Lentzen, Patrick Grysan, Santhana Eswara, Jean-Nicolas Audinot, Servane Contal, Johanna Ziebel, Cédric Guignard, Lucien Hoffmann, AlberTinka J. Murk, Arno C. Gutleb

**Affiliations:** 1 Environmental Research and Innovation (ERIN) Department, Luxembourg Institute of Science and Technology (LIST), 5 avenue des Hauts-Forneaux, L-4362 Esch-sur-Alzette, Luxembourg; 2 RIKILT- Institute of Food Safety, Wageningen UR, P.O. Box 230, NL-6700 AE Wageningen, The Netherlands; 3 Faculty of Medicine, University of Information Technology and Management in Rzeszow, Sucharskiego 2, 35-225 Rzeszow, Poland; 4 Centre for Radiobiology and Biological Dosimety, Institute of Nuclear Chemistry and Technology, Dorodna 16, 03-195 Warszawa, Poland; 5 Materials Research and Technology Department (MRT), Luxembourg Institute of Science and Technology (LIST), 5 avenue des Hauts-Forneaux, L-4362 Esch-sur-Alzette, Luxembourg; 6 Sub-department of Environmental Technology, Wageningen, The Netherlands; 7 IMARES Wageningen UR Institute for Marine Resources & Ecosystem Studies, P.O. Box 57, NL 1780 AB Den Helder, The Netherlands; 8 Current address: Norwegian Institute for Water Research (NIVA), Gaustadalléen 21, NO-0349 Oslo, Norway

**Keywords:** Intestinal co-culture, Mucus layer, Proteomics, Toxicology, Silver nanoparticles

## Abstract

**Background:**

The increased incorporation of silver nanoparticles (Ag NPs) into consumer products makes the characterization of potential risk for humans and other organisms essential. The oral route is an important uptake route for NPs, therefore the study of the gastrointestinal tract in respect to NP uptake and toxicity is very timely. The aim of the present study was to evaluate the effects of Ag NPs and ions on a Caco-2/TC7:HT29-MTX intestinal co-culture model with mucus secretion, which constitutes an important protective barrier to exogenous agents in vivo and may strongly influence particle uptake.

**Methods:**

The presence of the mucus layer was confirmed with staining techniques (alcian blue and toluidine blue). Mono and co-cultures of Caco-2/TC7 and HT29-MTX cells were exposed to Ag NPs (Ag 20 and 200 nm) and AgNO_3_ and viability (alamar blue), ROS induction (DCFH-DA assay) and IL-8 release (ELISA) were measured. The particle agglomeration in the media was evaluated with DLS and the ion release with ultrafiltration and ICP-MS. The effects of the Ag NPs and AgNO_3_ on cells in co-culture were studied at a proteome level with two-dimensional difference in gel electrophoresis (2D-DIGE) followed by Matrix Assisted Laser Desorption Ionization - Time Of Flight/ Time Of Flight (MALDI-TOF/TOF) mass spectrometry (MS). Intracellular localization was assessed with NanoSIMS and TEM.

**Results:**

The presence of mucus layer led to protection against ROS and decrease in IL-8 release. Both Ag 20 and 200 nm NPs were taken up by the cells and Ag NPs 20 nm were mainly localized in organelles with high sulfur content. A dose- and size-dependent increase in IL-8 release was observed with a lack of cytotoxicity and oxidative stress. Sixty one differentially abundant proteins were identified involved in cytoskeleton arrangement and cell cycle, oxidative stress, apoptosis, metabolism/detoxification and stress.

**Conclusions:**

The presence of mucus layer had an impact on modulating the induced toxicity of NPs. NP-specific effects were observed for uptake, pro-inflammatory response and changes at the proteome level. The low level of overlap between differentially abundant proteins observed in both Ag NPs and AgNO_3_ treated co-culture suggests size-dependent responses that cannot only be attributed to soluble Ag.

**Electronic supplementary material:**

The online version of this article (doi:10.1186/s12989-016-0117-9) contains supplementary material, which is available to authorized users.

## Background

Nanotechnology has brought about many advances in various fields from medicine and consumer products to environmental remediation. Nanotechnology has also raised many environmental and human health concerns due to the continuous increase in use and application of nanoparticles (NPs, all three dimensions in the order of 100 nm or less). Silver nanoparticles (Ag NPs) are commonly used in consumer products and numerous studies have shown size dependent effects, that differ from ionic Ag, in both animal models as well as in vitro cell culture models [1]. Among the reported effects are increased reactive oxygen species (ROS) levels, DNA damage and cell cycle arrest [2].

There is still a lack of knowledge about the fate and effects of Ag NPs on the gastrointestinal tract after oral exposure. This uptake route is possible via accidental ingestion during the production or due to their presence in water and in the food chain (due to their increased use in food preservation, food packaging material and water disinfection), therefore the gastrointestinal tract could be a target organ for Ag NP exposure [3, 4]. However, there are only a few studies dealing with adverse effects of NPs on the gastrointestinal epithelium [5–12]. The use of co-cultures and organo-typic culture systems has become more and more popular in toxicology over the last years due to the increased demand for more meaningful in vitro tests that can better mimic the in vivo situation, especially for the toxicity assessment of the increasing range of NPs [13]. In many different studies, co-culture systems have been proven to react in a more realistic way and to be more predictive of the in vivo response [6, 14, 15]. Caco-2 cells are widely used as a model for the intestinal epithelium in studies for drug permeability or nutrients and xenobiotics absorption and transport [16]. Caco-2 cells were also used in several studies to evaluate the effects of engineered NPs including Ag NPs [5, 7, 8, 10, 11]. However, the intestinal epithelium, apart from absorptive cells, also contains mucus-secreting cells (goblet cells) among others [17]. The mucus is a selective and dynamic barrier protecting against toxic agents, particulate matter and pathogens [18–20], while facilitating the exchange of nutrients, metabolites, water and gases. Mucus is continuously secreted, therefore particles etc. will have to move upstream to reach the epithelial cells [21]. Mucus is composed of a complex mixture of mucin molecules, lipids, proteins and other components that affect drug transport [20, 22]. It has been suggested that the mucus layer has effects on Fe bioavailability and absorption [23] and it represents an additional barrier to the transport of ions [19]. The mucus layer can trap NPs dependent on the NP size and surface charge [4]. The absence of the mucus layer, though, can potentially lead to over/under estimation of effects making the need for use of more physiologically relevant models evident.

The aim of the present study was to study the effects of different-sized Ag particles (Ag 20 and 200 nm) as well as AgNO_3_ as a source of ionic silver on an intestinal co-culture model which incorporates mucus for a more realistic simulation of the intestinal epithelium in vitro. The hypothesis is that the mucus layer provides a protective barrier against NPs and ions, and that mucin-NPs complexes modify the reactive surface and lead to a different uptake pattern of the NPs depending on particle size. The human colon cancer cells Caco-2/TC7 [24] as well as the human adenocarcinoma mucus-secreting cells HT29-MTX [25] were used in the presented intestinal model. These two cell lines represent the two major cell types (absorptive and mucus secreting cells) that are present in the intestinal epithelium in vivo. This model has been described before for iron bioavailability prediction studies [26] and drug absorption [27] including the evaluation of the efficiency of nanocarriers [28]. The metabolic activity of the cells was studied as a measure of cytotoxicity, reactive oxygen species generation and pro-inflammatory effects were studied on Caco-2/TC7, HT29-MTX cells alone and their co-culture. The effect of the Ag NPs and AgNO_3_ on cells in co-culture was studied at a proteome level by applying two-dimensional difference in gel electrophoresis (2D-DIGE) followed by Matrix Assisted Laser Desorption Ionization - Time Of Flight/ Time Of Flight (MALDI-TOF/TOF) mass spectrometry (MS) technique. This proteomic research can reveal key proteins and pathways that could be altered upon exposure to Ag NPs and AgNO_3_ and elucidate whether the observed changes can be related to the different sizes or ion release. In addition, NanoSIMS and TEM analyses were carried out to study potential NP uptake and intracellular localization in relation to particle size.

## Results

### Ag particle characterization in cell culture medium

**Fig. 1:**
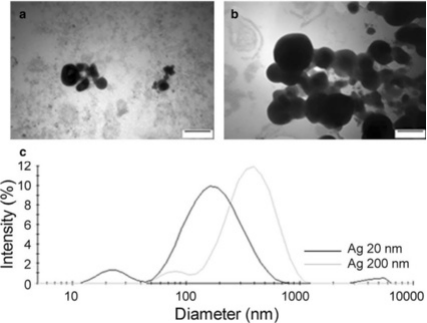
Particle characterization. Transmission electron microscopy (TEM) images of (**a**) Ag 20 nm and (**b**) Ag 200 mn. (**c**) Size distribution of the particles Ag 20 nm and Ag 200 nm in the cell culture medium with 10 % FBS at the highest exposure concentration used (100 mg/L). Scale bar is 100 nm

After suspension of the particles in full cell culture medium, containing 10 % FBS, both Ag 20 and 200 nm agglomerated and resulted in a broad size distribution with higher hydrodynamic sizes (Fig. 1, Table 1). The ζ potential for both particles in the FBS-containing cell culture medium was around -13 mV.

**Table 1:** Overview of particle characteristics. Main characteristics of the Ag particles studied and the ions released in cell culture medium containing 10 % FBS after 24 h at the highest concentration at which the cells were exposed to (100 mg/L)

Particles	Primary size (nm)	Hydrodynamic diameter (nm)	Z potential (mV)	Soluble Ag released (μg/L)
Ag 20 nm	20	129	-12.8	<10 (0.01 %)
Ag 200 nm	200	308	-13.9	< 10 (0.01 %)

The Z potential in MilliQ water was -14.2 and -11 for Ag 20 and 200 nm, respectively while the values in DMEM without phenol red and FBS were +3.5 (Ag 20 nm) and +0.5 mV (Ag 200 nm)

The soluble silver present in medium with FBS after 6 and 24 h of exposure to Ag NPs 20 and 200 nm was found to be less than 10 μg/L, which corresponds to less than 0.01 % release (Table 1).

### Alamar blue assay

**Fig. 2:**
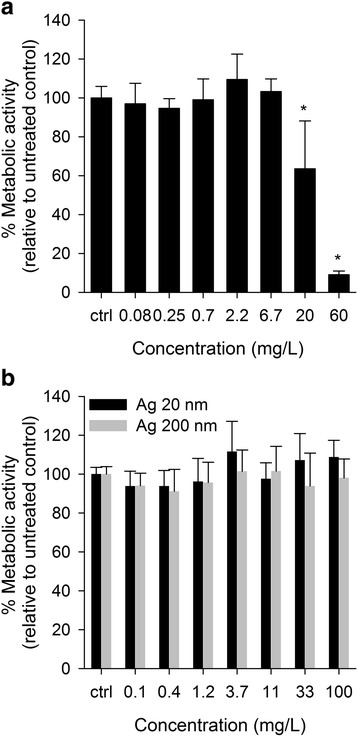
Effects on metabolic activity. Effects of (**a**) AgNO_3_ and (**b**) Ag 20 and 200 nm on the viability (determined as metabolic activity) of Caco-2/TC7 and HT29-MTX cells in co-culture at a 90:10 ratio. After 14 days in culture the cells were exposed for 24 h. Error bars represent the mean ± SD of 3 independent experiments performed in triplicate. * indicates significant differences of the treatments from the respective untreated controls (*P* < 0.05)

The viability of the cells in 90:10 co-culture (determined as metabolic activity) significantly decreased at concentrations higher than 20 mg/L of AgNO_3_ after 24 h of exposure (Fig. 2a). The co-culture was less affected by the treatment with AgNO_3_ compared to Caco-2/TC7 cells, however the differences were not statistically significant and therefore, only the results obtained with the cells in co-culture are shown. Neither Ag 20 nm nor Ag 200 nm induced cytotoxicity at any of the tested concentrations (0-100 mg/L) (Fig. 2b). Also no cytotoxicity was induced by the Ag particles in the Caco-2/TC7 and HT29-MTX cell lines cultured alone (data not shown).

### Intracellular reactive oxygen species formation

**Fig. 3:**
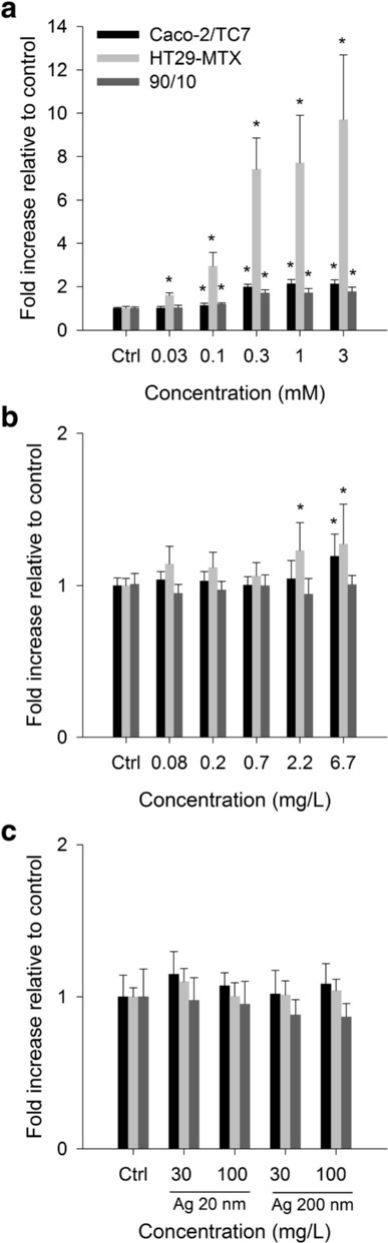
Intracellular reactive oxygen species formation. Effect of (**a**) H_2_O_2_ (positive control), (**b**) AgNO_3_ and (**c**) Ag 20 and 200 nm particles on the reactive oxygen species formation. After 14 days in culture Caco-2/TC7, HT29-MTX and the 90:10 co-culture were exposed for 2 h. Error bars represent the mean ± SD of 3 independent experiments performed in triplicate. Significant differences from respective untreated controls are marked with asterisks (* for *P* < 0.05)

Figure 3a shows a dose-dependent enhanced production of ROS in both cell lines as well as in the co-culture, upon exposure to the positive control H_2_O_2_ (0.03-3 mM). The highest levels of ROS were observed with the HT29-MTX cells upon exposure to the positive control. The response in the co-culture was lower than in Caco-2/TC7 and HT29-MTX alone and the lowest observed effect concentration was 0.03 mM for HT29-MTX and 0.1 mM for the Caco-2/TC7 cells alone as well as the co-culture. The Ag NPs and AgNO_3_ led to ROS production only in the Caco-2/TC7 and HT29-MTX mono-cultures with ROS levels of less than 15 % of the maximal levels induced by the positive control H_2_O_2._ In a cell free system the Ag particles did not lead to an increased fluorescence signal which was at the same levels as DCFH-DA in the medium alone (Additional file 1: Figure S5).

### IL-8 release

**Fig. 4:**
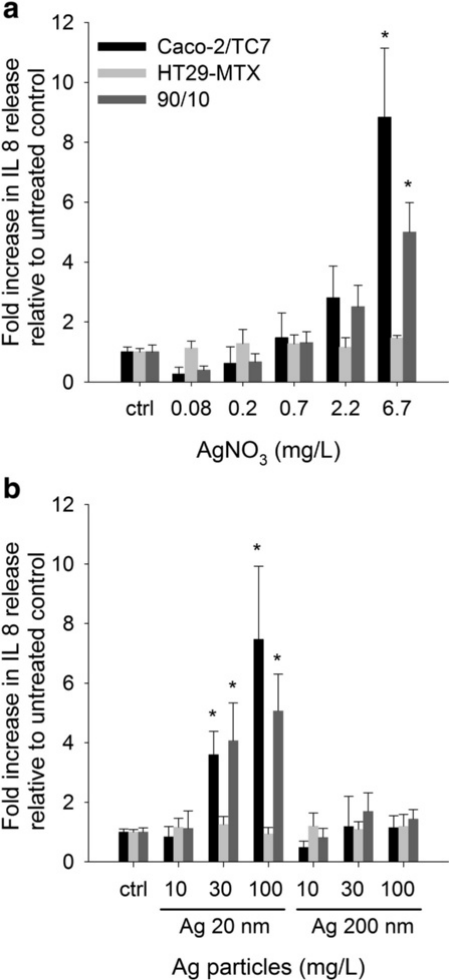
IL-8 release. IL-8 release in exposed Caco-2/TC7, HT29-MTX cells and their co-culture to (**a**) AgNO_3_ and (**b**) Ag 20 and 200 nm particles. Differentiated cells (after 14 days of culture) were exposed for 24 h. Error bars represent the mean ± SD of 3 independent experiments performed in duplicate. Significant differences from respective untreated controls are marked with asterisks (* for *P* < 0.05)

The pro-inflammatory effects of Ag NPs and AgNO_3_ were measured as secretion of interleukine-8 (IL-8) in the supernatant of exposed cells. A dose-dependent effect for AgNO_3_ in both Caco-2/TC7 as well as in the co-culture after 24 h of exposure was observed (Fig. 4a). The amount of IL-8 measured in the supernatant of exposed cells in co-culture was 2 times lower compared to the Caco-2/TC7 cells cultured alone at a concentration of 6.7 mg/L (highest AgNO_3_ concentration where no cytotoxic effects were observed). The HT29-MTX cells alone did not respond to stimulation by AgNO_3_. Increased levels of IL-8 were observed in the Caco-2/TC7 cells and in the co-culture upon exposure to Ag 20 nm NPs at concentrations above 30 mg/L. On the contrary, treatment with Ag 200 nm did not induce any statistically significant change. This shows a clear size-dependent effect (6- and 3.5-fold increase in IL-8 release compared to Ag 200 nm-exposed Caco-2/TC7 and cells in co-culture, respectively) (Fig. 4b).

### Nanoparticle uptake in the co-culture-NanoSIMS

**Fig. 5:**
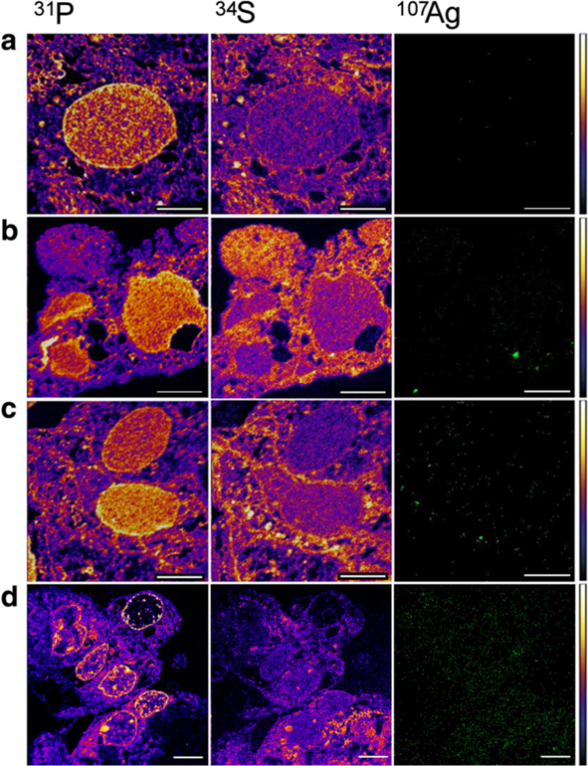
Elemental distribution of ^31^P, ^34^S and ^107^Ag (green) in 300 nm cuts. Caco-2/TC7:HT29-MTX cells in 90:10 co-culture were exposed to (**b**) Ag 20 nm (**c**) Ag 200 nm and (**d**) AgNO_3_ for 24 h while (**a**) represents the untreated control cells. Scale bar is 5 μm. The color scales indicated are for P and S images. For Ag images, the secondary ion intensity is expressed in green scale

In Fig. 5, the elemental distribution of ^31^P, ^34^S and ^107^Ag in cells in co-culture is shown. The elemental distribution of ^31^P, ^34^S was chosen in order to localize structures with high S and P content (nucleus, proteins etc.) as well as due to the high affinity of Ag for P and S. Intracellular presence of Ag was observed for both Ag 20 and 200 nm as well as AgNO_3_. After 24 h of exposure to 100 mg/L of particles, Ag from the Ag 20 nm NPs exposure (Fig. 5b) was found to be present in specific areas having a high content of sulfur or phosphorus. A high signal of Ag was also detected in the AgNO_3_-exposed cells.

### Proteomic analysis 2D-DIGE

At a non-cytotoxic concentration (1 mg/L) changes in relative abundance were found for 61 proteins upon exposure to Ag 20, 200 nm or AgNO_3_ compared to the untreated control in the co-culture model. In the AgNO_3_, Ag 20 nm and Ag 200 nm- treated cells in co-culture, 16, 50 and 6 proteins were altered, respectively (Table 2).

**Table 2:** Proteins with altered abundance. The cells in co-culture were exposed to the non-toxic concentration of 1 mg/L of Ag 20 nm NPs, Ag 200 nm NPs or AgNO_3_^a)^

Spot N°	Protein name	UniProt Ac. N°	UniProt ID	NCBI GI	Cov. %	MOWSE Score	p-value	Queries matched	Mr (Da)	pI (pH)	AgNO_3_ vs Control		Ag 20nm vs Control		Ag 200nm vs Control	
											Fold Change	t-test	Fold Change	t-test	Fold Change	t-test
	Cytoskeleton-associated proteins															
260	villin-1	P09327	VILI_HUMAN	194394237	40%	153	2.7e-010	40	93093	5.99	−1.13	0.351	−1.03	0.792	*1.55*	*0.047*
701	keratin, type II cytoskeletal 8	P05787	K2C8_HUMAN	4504919	64%	274	2.2e-022	38	53671	5.52	−1.29	0.451	*2.05*	*0.026*	−1.97	0.12
800	keratin, type II cytoskeletal 8	P05787	K2C8_HUMAN	4504919	67%	345	1.7e-029	43	53671	5.52	1.13	0.468	*1.7*	*0.002*	1.16	0.18
1083	keratin, type II cytoskeletal 8	P05787	K2C8_HUMAN	4504919	59%	412	3.4e-036	44	53671	5.52	*3.1*	*0.001*	*1.59*	*0.022*	1.1	0.598
841	cytokeratin 18 (424 AA)	P05783	K1C18_HUMAN	30311	77%	387	1.1e-033	46	47305	5.27	1.1	0.502	*1.49*	*0.029*	1.02	0.802
263	gelsolin isoform a precursor	B7Z9A0	B7Z9A0_HUMAN	221045118	35%	130	5.4e-008	34	83451	5.58	−1.1	0.751	*1.77*	*0.033*	1.33	0.218
984	actin, cytoplasmic 1	P60709	ACTB_HUMAN	14250401	72%	527	1.1e-047	38	41321	5.56	−1.1	0.114	**−1.74**	**0.004**	−1.05	0.282
993	actin, cytoplasmic 1	Q53G99	Q53G99_HUMAN	62897625	85%	642	3.4e-059	42	42080	5.37	−1.22	0.083	**−1.94**	**0.001**	−1.1	0.319
683	tubulin alpha-1B chain	P68363	TBA1B_HUMAN	119578453	60%	317	1.1e-026	30	46797	4.96	**−2.16**	**0.009**	−1.55	0.077	1.07	0.815
713	tubulin, beta 2C, isoform CRA_b	Q8N6N5	Q8N6N5_HUMAN	119608775	55%	272	3.4e-022	38	49250	4.88	**−3.8**	**0.003**	−1.22	0.331	1.32	0.267
538	dihydropyrimidinase-related protein 2	Q16555	DPYL2_HUMAN	4503377	62%	347	1.1e-029	40	62711	5.95	**−1.62**	**0.052**	1.44	0.08	1.44	0.117
	Oxidative stress-associated proteins															
1492	peroxiredoxin-6	P30041	PRDX6_HUMAN	4758638	89%	718	8.6e-067	46	25133	6.00	**−1.45**	**0.005**	**−1.25**	**0.036**	**−1.33**	**0.032**
627	protein disulfide-isomerase A3	P30101	PDIA3_HUMAN	220702506	58%	271	4.3e-022	38	55328	5.61	1.1	0.383	*2.01*	*0.004*	*1.38*	*0.038*
629	protein disulfide-isomerase A3	P30101	PDIA3_HUMAN	220702506	64%	795	1.7e-074	43	54541	5.61	1.34	0.18	*2.63*	*0.007*	1.42	0.106
635	protein disulfide-isomerase A3	P30101	PDIA3_HUMAN	220702506	63%	645	1.7e-059	44	54541	5.61	1.32	0.146	*2.53*	*0.003*	1.22	0.2
655	protein disulfide isomerase family A, member 3, isoform CRA_a	B3KQT9	B3KQT9_HUMAN	119597640	40%	81	0.0039	21	54454	6.78	*1.69*	*0.043*	1.1	0.247	−1.06	0.514
765	protein disulfide-isomerase A6	Q15084	PDIA6_HUMAN	1710248	63%	419	6.8e-037	38	46512	4.95	1.32	0.118	*1.52*	*0.04*	−1.11	0.618
769	protein disulfide-isomerase A6	Q15084	PDIA6_HUMAN	1710248	63%	467	1.1e-041	43	46512	4.95	1.25	0.104	*1.53*	*0.02*	1.05	0.757
767	glutathione synthetase	P48637	GSHB_HUMAN	4504169	70%	580	5.4e-053	50	52523	5.67	−1.08	0.367	*1.48*	*9E-04*	1.01	0.79
	Apoptosis-associated proteins															
1203	annexin A4	P09525	ANXA4_HUMAN	1703319	73%	453	2.7e-040	39	36088	5.84	−1.27	0.021	**−1.54**	**0.01**	−1.04	0.703
1208	annexin A4	P09525	ANXA4_HUMAN	1703319	69%	346	1.4e-029	37	36088	5.84	−1.16	0.434	−1.1	0.611	*1.88*	*0.009*
1226	annexin A4	P09525	ANXA4_HUMAN	1703319	73%	731	4.3e-068	42	36088	5.84	−1.24	0.119	**−1.63**	**0.006**	−1.06	0.676
1769	histidine triad nucleotide-binding protein 1	P49773	HINT1_HUMAN	227968190	94%	105	1.7e-005	18	13887	6.24	1.16	0.224	*1.36*	*0.013*	1.24	0.037
1773	programmed cell death protein 5	O14737	PDCD5_HUMAN	159163907	84%	217	1.1e-016	22	12911	9.85	*1.72*	*0.011*	1.03	0.896	−1.45	0.115
	Stress-associated proteins															
408	heat shock cognate 71 kDa protein isoform 2	Q53HF2	Q53HF2_HUMAN	24234686	57%	419	6.8e-037	39	53598	5.62	−1.03	0.896	*1.55*	*0.023*	−1.04	0.845
414	heat shock 70 kDa protein 1A/1B	P08107	HSP71_HUMAN	147744565	62%	248	8.6e-020	33	70294	5.48	*1.46*	*0.016*	*1.57*	*0.017*	1.1	0.493
429	heat shock 70 kDa protein 1A/1B	P08107	HSP71_HUMAN	147744565	56%	515	1.7e-046	45	70294	5.48	*1.33*	*0.01*	*1.61*	*3E-04*	1.28	0.269
438	heat shock 70 kDa protein 1A/1B	P08107	HSP71_HUMAN	147744565	51%	203	2.7e-015	31	70294	5.48	*1.54*	*0.034*	*2.04*	*0.021*	1.04	0.913
573	60 kDa heat shock protein, mitochondrial	P10809	CH60_HUMAN	31542947	53%	357	1.1e-030	43	61187	5.70	−1.03	0.884	*1.74*	*0.029*	−1.22	0.44
576	60 kDa heat shock protein, mitochondrial	P10809	CH60_HUMAN	31542947	45%	138	8.6e-009	30	61187	5.70	−1	0.86	*1.75*	*0.004*	−1.15	0.356
577	60 kDa heat shock protein, mitochondrial	P10809	CH60_HUMAN	31542947	57%	641	4.3e-059	47	61187	5.70	−1.04	0.889	*1.85*	*0.048*	−1.21	0.577
578	60 kDa heat shock protein, mitochondrial	P10809	CH60_HUMAN	31542947	72%	684	2.2e-063	49	61187	5.70	−1.04	0.869	*1.66*	*0.067*	−1.26	0.469
473	stress-induced-phosphoprotein 1	P31948	STIP1_HUMAN	5803181	54%	131	4.3e-008	29	68721	7.81	1.05	0.57	*2.02*	*0.002*	*1.37*	*0.012*
479	stress-induced-phosphoprotein 1	P31948	STIP1_HUMAN	5803181	58%	122	3.4e-007	28	68721	7.81	1.08	0.404	*2.03*	*0.003*	1.29	0.032
	Metabolism-associated proteins															
625	liver carboxylesterase 1	P23141	EST1_HUMAN	30749518	45%	133	2.7e-008	38	60692	6.06	−1.06	0.869	**−1.91**	**0.028**	−1.18	0.488
631	liver carboxylesterase 1	P23141	EST1_HUMAN	30749518	51%	109	6.8e-006	31	60692	6.06	−1.13	0.732	**−2.59**	**0.017**	−1.48	0.191
1543	glutathione S-transferase P	P09211	GSTP1_HUMAN	4504183	75%	716	1.4e-066	29	23569	5.43	−1.14	0.435	**−1.95**	**0.008**	−1.29	0.19
1569	glutathione S-transferase P	P09211	GSTP1_HUMAN	4504183	75%	681	4.3e-063	26	23569	5.43	−1.03	0.791	**−1.68**	**0.008**	−1.17	0.266
1866	fatty acid binding protein	P07148	FABPL_HUMAN	182356	91%	408	8.6e-036	21	14226	6.60	1.26	0.195	*1.88*	*0.008*	−1.07	0.706
1177	inorganic pyrophosphatase	Q15181	IPYR_HUMAN	11056044	77%	234	2.2e-018	29	33095	5.54	−1.16	0.332	*1.41*	*0.05*	1.2	0.366
1199	inorganic pyrophosphatase	Q15181	IPYR_HUMAN	11056044	68%	169	6.8e-012	25	33095	5.54	−1.03	0.975	*1.91*	*0.031*	1.26	0.309
1045	sialic acid synthase	Q9NR45	SIAS_HUMAN	12056473	76%	397	1.1e-034	39	40738	6.29	−1.15	0.548	*1.58*	*0.046*	1.37	0.109
1133	pyridoxal kinase	O00764	PDXK_HUMAN	119629883	45%	123	2.7e-007	24	42931	7.59	−1.06	0.702	**−1.7**	**0.008**	1.01	0.988
1320	purine nucleoside phosphorylase	P00491	PNPH_HUMAN	157168362	73%	244	2.2e-019	29	32758	6.71	−1.25	0.249	**−2.03**	**0.028**	−1.18	0.405
941	aspartate aminotransferase, cytoplasmic	P17174	AATC_HUMAN	4504067	72%	298	8.6e-025	39	46447	6.52	*1.85*	*0.039*	1.03	0.966	1.11	0.632
1938	polyubiquitin-C	F5H7Y5	F5H7Y5_HUMAN	228311825	80%	556	1.4e-050	24	17081	6.22	*1.36*	*0.016*	1.09	0.514	−1.19	0.268
185	elongation factor 2	P13639	EF2_HUMAN	4503483	37%	197	1.1e-014	38	96246	6.41	1.24	0.164	**−1.82**	**0.021**	1.15	0.595
200	elongation factor 2	P13639	EF2_HUMAN	4503483	46%	258	8.6e-021	45	96246	6.41	−1.15	0.164	**−1.67**	**0.009**	1.21	0.646
1734	peptidyl-prolyl cis-trans isomerase A	P62937	PPIA_HUMAN	1633054	93%	443	2.7e-039	23	18098	7.82	*1.78*	*0.05*	**−1.48**	**0.037**	−1.27	0.125
994	leukocyte elastase inhibitor	P30740	ILEU_HUMAN	13489087	56%	563	2.7e-051	39	42829	5.90	**−1.65**	**0.003**	**−2**	**0.001**	−1.25	0.147
1007	leukocyte elastase inhibitor	P30740	ILEU_HUMAN	13489087	52%	266	1.4e-021	27	42829	5.90	**−1.65**	**0.02**	**−1.75**	**0.009**	−1.39	0.151
1020	leukocyte elastase inhibitor	P30740	ILEU_HUMAN	13489087	56%	662	3.4e-061	39	42829	5.90	−1.22	0.024	**−1.32**	**0.005**	1.11	0.021
703	retinal dehydrogenase 1	P00352	AL1A1_HUMAN	21361176	52%	318	8.6e-027	45	55454	6.30	−1.28	0.064	**−1.62**	**0.01**	1.19	0.18
815	alpha-enolase	P06733	ENOA_HUMAN	203282367	78%	531	4.3e-048	48	47350	6.99	1.07	0.614	*1.92*	*0.008*	1.12	0.479
816	alpha-enolase	P06733	ENOA_HUMAN	203282367	80%	383	2.7e-033	45	47350	6.99	−1.03	0.803	*1.54*	*0.008*	1.05	0.737
1426	triosephosphate isomerase isoform 1	P60174	TPIS_HUMAN	4507645	97%	631	4.3e-058	51	26938	6.45	1.13	0.515	*1.95*	*0.025*	1.09	0.582
1443	triosephosphate isomerase isoform 1	P60174	TPIS_HUMAN	4507645	94%	830	5.4e-078	53	26938	6.45	−1.02	0.946	*1.92*	*0.049*	1.18	0.501
1461	triosephosphate isomerase isoform 1	P60174	TPIS_HUMAN	66360365	55%	171	4.3e-012	26	26938	6.45	**−1.56**	**0.033**	−1.08	0.667	−1.13	0.494
1008	fructose-bisphosphate aldolase C	P09972	ALDOC_HUMAN	61680382	61%	138	8.6e-009	23	37940	6.67	−1.23	0.047	−1.07	0.334	*1.35*	*0.003*
	Others															
1357	chloride intracellular channel protein 1	O00299	CLIC1_HUMAN	14251209	81%	322	3.4e-027	31	27248	5.09	1.2	0.107	**−1.55**	**0.009**	−1.2	0.064
1370	chloride intracellular channel protein 1	O00299	CLIC1_HUMAN	14251209	75%	506	1.4e-045	38	27248	5.09	−1.04	0.808	**−1.65**	**0.013**	−1.22	0.213

^a)^The spot number; protein name; UniProt accession number; UniProt ID; NCBI accession number; sequence coverage; MOWSE score; p-value relative to the MASCOT identification; number of queries matched; theoretical molecular weight (expressed in Da) and isoelectric point (expressed in pH units); the fold change and relative t-test value for co-cultures treated with AgNO_3_, Ag 20 nm NPs and Ag 200 nm NPs, respectively, are reported for each protein. Italics = protein is more abundant; Bold = protein is less abundant.

**Fig. 6:**
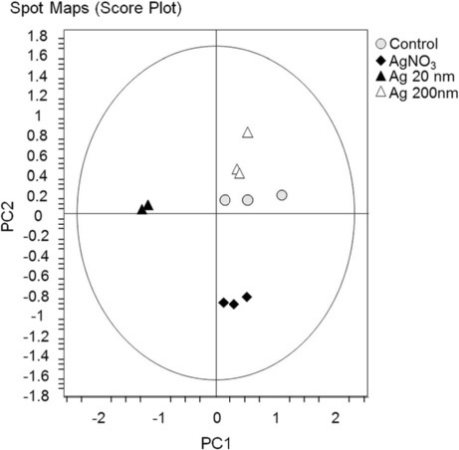
Principal component analysis of differentially expressed proteins. Caco-2/TC7:HT29-MTX cells in 90:10 co-culture were exposed to 1 mg/L of Ag 20 nm, Ag 200 nm or AgNO_3_ for 24 h

The 61 differentially expressed proteins were used to cluster the samples by principal component analysis (PCA) compared to the negative control (Fig. 6). Co-cultures treated with Ag 200 nm clustered very close to the negative control, while the Ag 20 nm and AgNO_3_ treatments showed bigger differences in the proteome compared to the untreated cells or the Ag 200 nm-treated cells. The Ag 20 nm and AgNO_3_-induced proteome changes clustered far away from each other. The hierarchical clustering, based on these proteins provided the same overall picture (Additional file 1: Figure S7).

Proteins with significant changes were classified in 6 main categories according to their function (Table 2) involving: cytoskeleton organization and cell cycle regulation, redox regulation, apoptosis, stress response, detoxification/metabolism regulation or in “other functions” when the previous categories did not fit.

The only one protein that was found to be changed by Ag 20 and 200 nm as well as AgNO_3_ exposure was peroxiredoxin-6 that is involved in redox regulation of the cell.

**Fig. 7:**
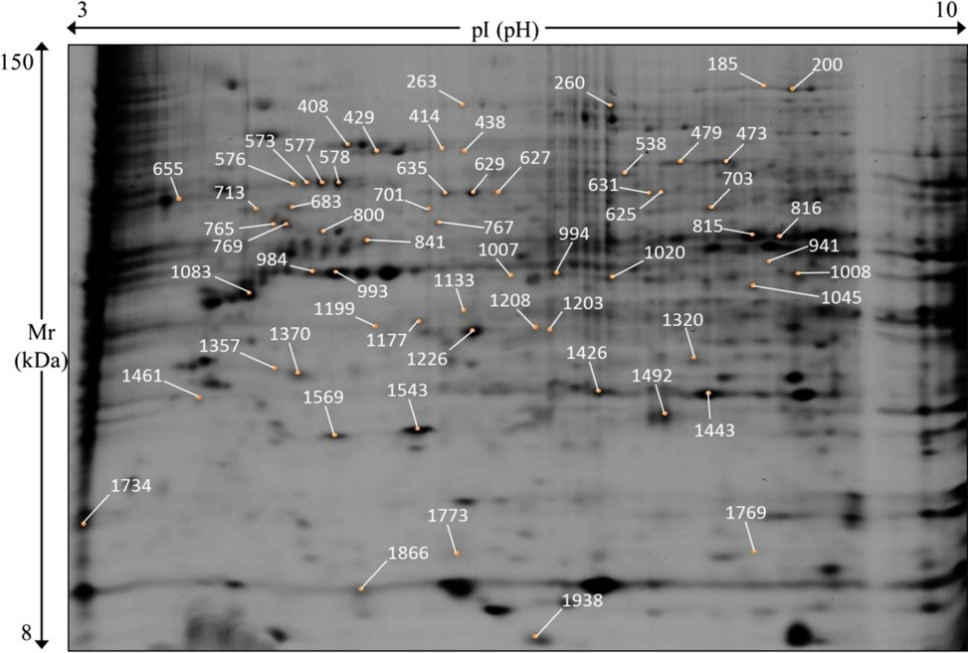
A representative 2D-DIGE gel. Caco-2/TC7:HT29-MTX cells in 90:10 co-culture were exposed to 1 mg/L of Ag NPs 20 nm, 200 nm or AgNO_3_ for 24 h prior to protein extraction. Cytosolic proteins were separated in first dimension on 24 cm strips, pH 3–10 non-linear and in second dimension on a 12.5 % polyacrylamide precast gel. Yellow dots indicate picking location on the gels. For each spot the relative spot number is reported

Figure 7 is a representative image showing the picking location for proteins which were altered upon exposure to Ag 20 nm, Ag 200 nm or AgNO_3_. The fold change and additional information of all differentially abundant proteins in the different treatment groups can be found in Table 2. Detailed information on protein identification is presented in the additional files (Additional file 1: Table S1).

## Discussion

Ag NPs with their increased use in consumer products are likely to reach the environment and humans through either direct or indirect exposure. Although an important uptake route for humans is via ingestion, the effects of NPs and their fate in the gastrointestinal tract are largely unknown. This study describes the evaluation of the uptake, intracellular localization and effects of Ag NPs and AgNO_3_ on a gastrointestinal co-culture model simulating the epithelium incorporating a mucus layer. The aim of this study was to incorporate the mucus in order to obtain a more realistic in vitro estimation of the toxic potential of silver particles, as mucus can act as a barrier that impedes, or at least reduces, the interaction between the cells and the NPs that can be dependent on particle size.

In the gastrointestinal epithelium the mucus layer provides a protective barrier against pathogens, digestive enzymes and damage [26]. The staining performed showed that the mucus layer was formed after 14 days in culture and covered the surface of the cell monolayer at the lowest ratio of HT29-MTX cells present (90:10) (Additional file 1: Figures S1-S3). The concentration of goblet cells in the small intestine and under healthy conditions is approximately 10 % [17] and for that reason this ratio was chosen for the study. Low amounts of mucus can be produced by Caco-2 cells under normal conditions, however this was not observed in our study with the staining techniques used [29].

The NanoSIMS analysis revealed a cellular uptake of Ag by the cells in co-culture exposed to both 20 and 200 nm Ag particles which was further confirmed by ICP-MS quantification (Additional file 1: Table S2). The TEM analysis (Additional file 1: Figure S6) showed internalization of particles close to the cell membrane that further confirms the NanoSIMS data. In the case of Ag 200 nm exposed cells, particles were particularly found in the brush border. However, according to NanoSIMS analysis, Ag was observed in proximity of the nuclei*,* supporting the idea that “hot spots” could be mainly represented by ions, rather than NPs.

Interactions with serum proteins are to be expected and it has been shown that the fetal bovine serum (FBS) content of the medium influenced the extent of NPs’ uptake and toxic effects. In medium with 1 % FBS a concentration and size-dependent cytotoxicity of SiO_2_ NPs was observed while no cytotoxicity was reported in the 10 % FBS-containing medium [30]. In our study 10 % FBS was used which is the minimum content required for optimal growth and function of the Caco-2/TC7 and HT29-MTX cells. It has been reported that the rapid formation of protein corona led to an increase in the particle attachment to the cell surface and uptake and the particle type, surface functionalization and size affect the formation and composition of the corona [31]. The extent of Ag NPs’ surface interaction with the proteins is still unknown but complexation processes are to be expected that could be size-dependent. The zeta potential of both particles in culture medium with 10 % FBS was very similar and slightly negatively charged (-13 mV). Therefore, the differences in uptake observed in our study cannot be explained solely by the charge differences of the particles. The proteins present in the cell culture medium containing FBS differ significantly from the proteins found in the biological fluids in vivo, therefore the uptake, bioavailability and translocation of the particles may differ in vivo. A study using an in vitro model for gastrointestinal digestion showed that Ag NPs form clusters after gastric digestion that disintegrate resulting in the reappearance of particles after intestinal digestion in the presence of proteins suggesting that NPs can reach the intestinal epithelium [32].

Although oxidative stress is one of the proposed mechanisms for NP toxicity, the studied Ag NPs did not lead to a significant increase in intracellular ROS formation which is in agreement with previous studies using Caco-2 [5] and BEAS-2B cells [33]. The cells in co-culture were less responsive to oxidative stress induced by H_2_O_2_ compared to Caco-2/TC7 cells alone. It has been previously shown that the mucus layer has ROS scavenging abilities and resists to ROS attack while low levels of ROS increase the barrier protection by increasing the mucus layer thickness [34]. Therefore, it seems reasonable to hypothesize that the presence of the mucus layer provides protection against ROS damage.

A significant size-dependent increased IL-8 release was observed with the smaller Ag 20 nm eliciting an 8 and 4 times higher inflammatory response compared to the larger Ag 200 nm in Caco-2/TC7 cells alone and the co-culture, respectively. This is in accordance with previous findings using the same set of NPs where only 20 nm Ag NPs up-regulated the IL-8 expression [35]. A possible explanation for this effect could be that bigger particles have different transport rates and are better retained in the mucus layer than smaller ones or ions that may thus cross the mucus layer and reach the cells faster and induce stronger effects. This could also mean that the protective effect of the mucus against AgNO_3_ could be rather limited. This correlates with the higher Ag concentrations (2.5 times higher) found for Ag 200 nm particles compared to Ag 20 nm in the co-culture while the Ag signal observed in NanoSIMS was similar for both treatments suggesting that the bigger-sized particles are trapped in the mucus layer or are moving at a slower rate. Moreover, according to the TEM images Ag particles from the Ag 200 nm exposed cells are present in the brush border. A size dependency in the transit rate of NPs has been previously shown with smaller-sized particles showing a higher transit rate [29]. Thus, the immobilization of the bigger sized particles due to size exclusion by the mucus could result to reducing their interaction with the cellular membranes, the minor toxicity, low ROS levels and IL-8 release and decreased alterations at the proteome level observed in this study.

In order to have a comprehensive understanding of changes that occur upon Ag 20, Ag 200 nm and AgNO_3_ exposure in the co-culture model, a proteomic study was included. The biggest changes in protein expression were induced by Ag 20 nm (50 differentially regulated proteins) followed by AgNO_3_ (16 modified proteins), while treatment with Ag 200 nm NPs only induced changes to 6 cytosolic proteins.

Ag 20 nm triggered an up-regulation of the cytoskeleton proteins cytokeratin 8 (CK8) and cytokeratin 18 (424 AA) (CK18) that are essential for the integrity of the epithelial cells [36]. They are co-expressed in a variety of tissues including the gastrointestinal tract [37] playing an important role in maintaining barrier function under stress. CK8/CK18 have been reported to be involved in IL-6 mediated barrier protection [38]. In addition, actin, cytoplasmic 1 (ACTB) that is essential for maintaining the epithelial integrity and regulating the structure of tight junctions [39, 40] was down-regulated in Ag 20 nm-exposed cells while higher levels were found for the liver type fatty acid-binding protein-1 (FABPL), that regulates absorption and transport of fatty acids [41] and has been reported to be a marker of intestinal tissue injury [42]. Only the exposure to Ag 20 nm led to lower levels of chloride intracellular channel protein 1 (CLIC1) that is expressed in the apical part of columnar epithelia including the small intestine [43] and has been suggested to be associated with actin cytoskeleton [44]. It has been previously shown that CLIC1 is involved in cell cycle regulation and cell division and the over-expression of CLIC1 led to the inhibition of the proliferation of gastric cells and apoptosis induction [45]. In addition, the role of CLIC1 as a sensor of cell oxidation has been previously reported [46], which could suggest protection against apoptosis or response to oxidative conditions. The paracellular permeability, however, was not changed upon exposure to Ag NPs as measured with a paracellular permeability marker (lucifer yellow) and transepithelial electrical resistance (TEER) (Additional file 1: Figure S4).

Furthermore, higher levels of sialic acid synthase (SAS, also known as N-acetylneuraminate synthase) were observed in Ag 20 nm-treated cells. Sialic acids may have a structural role due to their presence in the outer surface of the cell and their negative charge and they are part of binding and recognition sites for pathogens and toxins [47] as well as they may mask recognition sites such as antigenic sites [48]. In addition, sialic acids are also localized in mucus glycoprotein and they contribute to the high viscosity of the mucus barrier and are of high importance for the maintenance of mucosal integrity [49]. An increased mucus production has been previously observed upon inflammatory conditions [29] and the higher levels of SAS observed in our study can be related to changes in mucus production in response to the increased IL-8 levels. The increased IL-8 levels observed in our study upon Ag 20 nm and AgNO_3_ and exposure can also be related to the changes observed in purine nucleoside phosphorylase (PNP) and peptidyl-prolyl cis-trans isomerase A (PPIA), also known as cyclophilin A (CypA), respectively. PNP deficiency has been associated with immune dysfunction [50, 51]. CypA can be released in the presence of inflammatory stimuli and it has been involved in several diseases (e.g. cardiovascular and inflammatory diseases, cancer) and plays a role in the regulation of infection and replication of several viruses [52]. CypA overexpression has been related with increased IL-8 levels and proliferation [53] as well as increased drug resistance [54].

Both Ag 20 nm (less abundant) and Ag 200 nm treatment (more abundant) led to altered levels of Annexin A4 (ANXA4), a key apoptosis regulator, that was suggested to be an early marker of apoptosis [55]. In the intestine, ANXA4 is present in both mature villus enterocytes (along the basolateral membrane) and goblet cells [56]. Moreover, higher levels of histidine triad nucleotide-binding protein 1 (HINT1) in Ag 20 nm treated cells were also observed that is involved in apoptotic processes and may also have tumor suppressor functions [57]. These results suggest that Ag NPs treatment affects apoptotic signaling in cells. The balance in cell proliferation and apoptosis is essential in the gastrointestinal epithelium for the maintenance of the normal function as a barrier [58].

A clear particle-size effect on the proteins that were differentially expressed was seen, that was also different from the pattern observed for AgNO_3,_ which is in accordance with a recent study showing that particles of different sizes regulate different sets of proteins [12]. This is particularly visible from the multivariate analysis that was performed on the set of altered proteins (Fig. 6): the PCA clearly showed that Ag 200 nm treated co-culture cluster together with the untreated cells, while AgNO_3_-treated and Ag 20 nm-treated co-cultures cluster away from the control and from each other. This could mean that the toxicity at a protein level is different for particles and soluble Ag (AgNO_3_) and that different sized particles induce different effects. Similar findings have been observed in a proteomic study of the plant *Eruca sativa* after exposure to either ionic or particulate silver indicating that the effects of Ag NPs are not due to ion release [59]. These results are in agreement with the IL-8 release findings and with a recent study with LoVo human colon cancer cells showing that more proteins were differentially regulated after exposure to Ag 20 nm than Ag 100 nm NPs regulating different sets of proteins [12]. Also in HepG2 cells exposed to the same Ag 200 nm particles, only minor changes in gene expression were found [35].

Ag 20 nm altered the expression of proteins that play an important role in xenobiotic metabolism probably compromising the detoxification process. The protein liver carboxylesterase 1 (CES1), responsible for the hydrolysis and metabolism of endogenous and exogenous compounds [60], and glutathione S-transferase P (GSTP1) were found to be less abundant in the Ag 20 nm-treated cells in co-culture. GSTP1 belongs to the family of phase II detoxification enzymes, responsible for the metabolism of xenobiotics and secondary metabolites/by-products of oxidative stress [61].

Normally cells have a defense system against oxidative stress with a variety of enzymes being involved. Both Ag NPs treatments and AgNO_3_ triggered a down-regulation of peroxiredoxin-6 (PRDX6, the only overlap observed for all treatments) that is a member of peroxiredoxins and is involved in redox regulation of the cell and protection against oxidative injury [62]. A previous suppression of PRDX6 led to increased ROS levels and apoptosis of cancer cells [63]. This implies that both Ag NPs and ions lead to a compromised oxidative defense system. Ag 20 nm activated proteins related to redox homeostasis. Increased levels of protein disulfide isomerases A3 and A6 (PDIA3 and PDIA6) were observed in Ag 20 nm-treated cells. Thus, the increased levels observed could counteract the effects induced by oxidative stress such as protein misfolding or damage. In addition, Ag 20 nm treatment led to increased levels of glutathione synthetase (GSS) that is critical for the synthesis of glutathione (GSH), a major anti-oxidant and detoxification agent [64] that provides defense against metabolite toxicity [65]. Therefore, these results indicate that both Ag NPs and AgNO_3_ but mainly Ag 20 nm, lead to the activation of mechanisms in order to deal with oxidative stress and maintain cellular homeostasis.

The list of altered proteins triggered by Ag NPs was subjected to KEGG enrichment analysis (performed with the EDA module in the Decyder software), with the aim of identifying altered cellular pathways. The analysis highlighted that a number of pathways seems to be affected by the treatment and in particular these are involved, along with metabolic pathways, oxidative stress pathways and protein processing pathways, in pathways related to pathogen invasion (e.g antigen presenting pathway and legionellosis). These pathways contribute to the inflammatory responses (e.g. IL-8 release) and suggest that the cell might detect the NPs as a pathogen. It has to be noted that the number of proteins included in the KEGG enrichment analysis was quite limited, thus these results should be carefully considered and validated with further studies.

In the current study, the proteomic results revealed an altered expression of proteins related to the maintenance of the redox balance of the cell, protection against oxidative damage and apoptosis as well as tissue damage and adaptation. The mechanisms of intestinal adaptation upon several internal and external stimuli involve altered expression levels of carrier proteins, changes in barrier permeability etc. [66] and proteins involved in the maintenance of the balance between cell proliferation and apoptosis which is a physiological event in the gastrointestinal epithelium. Our results revealed that Ag 20 nm NPs could result in a compromised intestinal barrier integrity and function as was shown with the increased levels of IL-8 as well as the altered levels of proteins that have been reported to be involved in intestinal injury and adaptation (e.g. ANXA4 and villin, CK8/CK18, gelsolin, ACTB, IPYR, FABPL, SAS) that could have implications on the physiological processes occurring in the gastrointestinal epithelium such as normal nutrient absorption and transport as well as protection against pathogens and xenobiotics. In addition, Ag NPs 20 nm led to an up-regulation of several proteins (GSTP1, CES1, GSS) that together with the multidrug efflux transporters are involved in the defense of the cells against xenobiotics and metabolites in several tissues. Certain NPs can inhibit multidrug efflux transporters [67, 68] and this could possibly result in up-regulation of compensation mechanisms.

## Conclusions

The co-culture model represents a more physiological and relevant in vivo-like model compared to the Caco-2 cells alone, with the presence of mucus which has an impact on modulating the induced toxicity of NPs in a size dependent manner. The mucus layer presents a mechanical barrier mostly towards bigger sized particles reducing their interaction with the cellular membrane and subsequently leading to minor toxicity, reduced oxidative stress, IL-8 release and proteomic alterations compared to Ag 20 nm particles and AgNO_3_. The proteomic results revealed that Ag NPs 20 nm regulated different sets of proteins with a distinct pattern of cellular responses compared to Ag 200 nm and AgNO_3,_ indicating a different mode of action with effects being particle and size-dependent. The changes observed in the proteome level and the increased IL-8 levels indicate that Ag NPs trigger a pathogen-like response and the regulation of proteins responsible for the maintenance of the intestinal barrier function and integrity. Further research should elucidate the uptake mechanisms in the co-culture for different sized particles and ions, the role of mucus on the transit rate of the different-sized NPs and the mechanism leading to increased inflammatory response.

## Methods

### Ag NPs and chemicals

Ag NPs (20 and 200 nm) were obtained from PlasmaChem GmbH (Berlin, Germany). Silver nitrate (AgNO_3_) was purchased from VWR (Leuven, Belgium) and Alcian blue, H_2_O_2_, Toluidine blue, 2′,7′-dichlorofluorescin diacetate (DCFH-DA), osmium tetroxide (OsO_4_), glutaraldehyde, resazurin sodium salt and the epoxy resin embedding kit from Sigma Aldrich (Bornem, Belgium). IL-8 kit was obtained from ENZO Life Sciences BVBA (Zandhoven, Belgium).

### Ag NPs dispersion protocols

Ag NP stock solutions were prepared as previously described [69, 70]. Briefly, the Ag NPs were suspended in 5 % DMSO in sterile Milli-Q water (Millipak Express, Millipore) at a concentration of 2 mg/mL and sonicated on ice for 3 min using a UP200S probe ultra sonicator (0.5 cycle, 30 % amplitude, Hielscher, Germany). Stocks were always prepared fresh prior to each experiment. The characteristics of the NPs in different culture media can be found in previous studies [69–71].

### Ag NPs characterization in medium

Dynamic light scattering and zeta (ζ) potential measurements of NPs in solution were carried out with a nanoZetasizer (Malvern Instruments Ltd, UK). The stocks were added to the full cell culture medium containing 10 % FBS in order to achieve the highest concentration at which the cells were exposed (100 mg/L).

### Cell culture

The human colon cancer Caco-2 cell line sub-clone TC7 (Caco-2/TC7) was a generous gift from Monique Rousset (Nancy University, France). HT29-MTX cells were kindly provided by T. Lesuffleur (INSERM UMR S 938, Paris, France). Phosphate Buffer Saline (PBS) and heat-inactivated Fetal Bovine Serum (FBS) were all obtained from Invitrogen (Merelbeke, Belgium).

Both cell lines were maintained in Dulbecco’s Modified Eagle Medium-Glutamax (DMEM-Glutamax, Invitrogen) supplemented with 10 % fetal bovine serum, 1 % non-essential amino acids and 1 % penicillin/streptomycin solution (at 37 °C in a 10 % CO_2_ humidified incubator). The medium was replaced every other day and cells were split upon confluency with Trypsin-EDTA. In order to ensure full differentiation of cells, experiments were carried out 14 days post seeding.

### Metabolic activity assay (Alamar Blue) as a measure of cytotoxicity

The single cell cultures of Caco-2/TC7, HT29-MTX and their co-culture at a 90:10 ratio were seeded at a concentration of 1.2x10^5^ cells/mL in 12-well plates and were grown for 14 days at 37 °C in a 10 % CO_2_ humidified incubator. After 14 days the medium was discarded and new medium containing increasing concentrations of the Ag NPs (0-100 mg/L) or AgNO_3_ (0-60 mg/L) was added (serially diluted in complete medium). For the cell viability assay for AgNO_3_ a maximum concentration of 60 mg/L was chosen based on preliminary experiments, while the maximum concentration chosen for the preliminary screening on cell viability for Ag particles was 100 mg/L. After 24 h of exposure, the medium containing the Ag NPs or AgNO_3_ was discarded and replaced with 1 mL of 500 μM resazurin per well. Resazurin is a cell permeable, non-fluorescent compound that is reduced by metabolically active cells into the fluorescent resorufin. After 1.5 h of incubation at 37 °C in a 10 % CO_2_ humidified incubator in the dark, fluorescence was measured at an excitation wavelength of 530 nm and emission wavelength of 590 nm (Synergy 2, BioTek Instruments, Inc.). The reported metabolic activity is expressed relative to the untreated group, which was set at 100 %.

For the following experiments, the concentrations were chosen based on the cell viability results. The highest concentrations that did not result in cell viability reduction are shown.

### Measurement of intracellular reactive oxygen species formation

The levels of reactive oxygen species (ROS) were evaluated with the use of a non-fluorescent probe (DCFH-DA) that once inside the cell it is deacetylated by cellular esterases into the non-fluorescent DCFH. When ROS are present DCFH is oxidized into the highly fluorescent DCF. The assay was optimized for the DCFH-DA concentration and the exposure time. The highest response to H_2_O_2_ and Ag was observed after 2 h of exposure and therefore this time point was chosen. Single cell culture of Caco-2/TC7 and HT29-MTX and their co-culture at a 90:10 ratio were seeded at a concentration of 1.2x10^5^ cells/mL in 12-well plates and were grown for 14 days at 37 °C in a 10 % CO_2_ humidified incubator. After 14 days the medium was discarded and replaced with 1 mL of 150 μM DCFH-DA per well. After 1 h of incubation at 37 °C in a 10 % CO_2_ humidified incubator in the dark, the dye was removed, the monolayers were washed with PBS and increasing concentrations of NPs or AgNO_3_ were added in the wells (serially diluted in cell culture medium). The highest concentration used for AgNO_3_ was 6.7 mg/L due to increased cytotoxicity. After 2 h, fluorescence was measured at an excitation wavelength of 480 nm and emission wavelength of 530 nm (Synergy 2, BioTek Instruments, Inc.). As a positive control, 0.01 % of H_2_O_2_ was included in every plate.

### Measurement of IL-8 release

Caco-2/TC7, HT29-MTX cells or cells in co-culture at a 90:10 ratio were seeded at a concentration of 1.2x10^5^ cells/mL in 12-well plates and were grown for 14 days at 37 °C in a 10 % CO_2_ humidified incubator. The medium was renewed every other day and after 14 days the medium was discarded and new medium containing increasing concentrations of the Ag NPs (0-100 mg/L) or AgNO_3_ (0-6.7 mg/L) was added (serially diluted in complete medium). The highest concentration used for AgNO_3_ was 6.7 mg/L due to increased cytotoxicity. At the end of the exposure period the supernatants were collected and stored at -80 °C until analysis. The amount of IL-8 released upon exposure to Ag 20, Ag 200 nm or AgNO_3_ for 24 h was evaluated with an enzyme-linked immunosorbent assay (ELISA) kit (Assay Designs/ENZO Life Sciences, Zandhoven, Belgium) according to the manufacturer’s instruction protocol.

### Total soluble Ag release over time (Ultrafiltration and ICP-MS)

The total amount of soluble Ag ionic species released from the Ag 20 and 200 nm particles in the cell culture medium was evaluated as previously described [69]. Briefly, 2 mL of exposure medium (medium + Ag NPs at the highest working concentration of 100 mg/L or only medium in the absence of cells) were taken after 6 and 24 h of incubation at 37 °C in a 10 % CO_2_ humidified incubator in the dark and were centrifuged for 40 min at 4000 g using centrifugal filter devices with 3 kDa cut-off (Amicon ultra-4, Millipore, Ireland). The ultra-filtrates were evaluated for the total soluble Ag content by Inductively Coupled Plasma Mass Spectrometry (ICP-MS) (Elan DRC-e, Perkin Elmer, Waltham, MA, USA) as previously described [69, 72].

### Ag NP uptake evaluation in the co-culture (NanoSIMS50)

The co-culture of Caco-2/TC7 and HT29-MTX at a 90:10 ratio was seeded in 12-well plates and after 14 days in culture the cells were exposed to 100 mg/L Ag 20 and 200 nm and 20 mg/L AgNO_3_ for 24 h. The cells were washed with PBS, detached with a cell scraper and transferred into eppendorf tubes. The settled cell pellets were fixed with 5 % glutaraldehyde in PBS overnight. Glutaraldehyde was removed and cells were washed with PBS. They were then post fixed with 1 % OsO_4_ in milliQ water for 2 h. After an additional washing step with PBS the cell pellets were placed in agar blocks (1 % agar), they were dehydrated with increasing acetone concentrations (30, 50, 70, 90 and 100 % acetone), and they were finally embedded in epoxy resin (Epon 812 substitute) in molds (easy molds, Ted Pella, Inc). Samples were cut to 300 nm semi-thin sections (Leica Ultracut UCT, Le Pecq Cedex, France) and deposited on silicon wafers (Siltronix, Archamps, France) for SIMS analysis. The NanoSIMS 50 [69, 73, 74] (Cameca, Courbevoie, France) analyses were performed by scanning of the surface (40 × 40 μm^2^ and 20 × 20 μm^2^) with a primary Cs^+^ ion. The impact of the primary beam was 16 keV with an intensity range of 1.0–0.8 pA. Images were recorded in a pixel format of 256 × 256 image points with a counting time of 20 ms per pixel. The probe-working diameter was estimated in the range of 80–100 nm. The secondary negative ions recorded simultaneously were: ^12^C^14^N^-^ (m = 26.00307 amu), ^31^P^-^ (m = 30.97376 amu), ^34^S^-^ (m = 33.96786 amu) and ^107^Ag^-^ (m = 106.90486 amu).

### Cellular Ag content determination

Caco-2/TC7, HT29-MTX cells or cells in co-culture at a 90:10 ratio were seeded at a concentration of 1.2×10^5^ cells/mL in 12-well plates and were grown for 14 days at 37 °C in a 10 % CO_2_ humidified incubator. The medium was renewed every other day and after 14 days the medium was discarded and new medium containing 100 mg/L Ag NPs 20 and 200 nm or 20 mg/L AgNO_3_ was added. In addition, a treatment of the cells in co-culture with 1 mg/L Ag 20 and 200 nm or AgNO_3_ was included. At the end of the exposure the cells were washed with PBS, they were detached with Trypsin-EDTA and the cell pellet was collected after centrifugation at 1500 rpm for 5 min. The samples were digested in 3 mL demineralized water with 1.75 mL HNO_3_ (min 67 % for trace analysis, LGC Standards, Germany) and 0.75 mL H_2_O_2_ (30 % Suprapur, Merck, Germany). Then the volume was completed to 10 mL with demineralized water and the obtained solution was diluted 3 times with 1 % HNO_3_ before analysis. Silver was analyzed by Inductively Coupled Plasma Mass Spectrometry (ICP-MS).

### Proteomic approach

The co-culture of Caco-2/TC7 and HT29-MTX cells at a 90:10 ratio was seeded in cell culture flasks (Nunclon, 175 cm^2^) at a concentration of 2.9 × 10^5^ cells/mL (30 mL/flask) and were grown for 14 days at 37 °C in a 10 % CO_2_ humidified incubator with a medium change every other day. After 14 days the medium was discarded and replaced by new medium containing 1 mg/L Ag 20, Ag 200 nm or AgNO_3_ for 24 h. This dose was selected in order to ensure that cytotoxicity would not affect the results. Four replicates per treatment were used including untreated/solvent control (0.25 % DMSO). At the end of the exposure period, the medium was removed and the monolayers were washed twice with 7 mL ice-cold PBS. Five mL of ice-cold PBS were added and the cell monolayers were detached using a cell scraper and transferred in a 15 mL falcon tube. After centrifugation for 5 min at 1500 rpm at 4 °C (Beckman TM Allegra 64R Beckman Coulter, CA, U.S.) the supernatant was removed and the cells were stored at -20 °C until protein extraction.

### Protein extraction

The cells were re-suspended in a lysis buffer (100 mM PIPES, 70 mM NaCl) supplemented with a protease inhibitor cocktail (Protease inhibitor mix, GE Healthcare, Little Chalfont, UK). The cellular pellets were disrupted by the use of the French Press cell disruptor (Thermo Electron Corporation, MA, U.S.) at a pressure of 200 psi. Total cellular lysates were collected and stored on ice. Subsequently, the lysate was pelleted at 2,100 rpm for 15 min at 4 °C using the Beckman TM Allegra 64R centrifuge on a F1010 rotor (Beckman Coulter, CA, U.S.) in order to remove nuclei from the supernatant.

The supernatant (containing all cellular fractions excluding the nuclei) was centrifuged at 20,000 rpm during 20 min at 4 °C in Beckman TM Optima TM L90K ultracentrifuge on a 45Ti rotor. This pellet consisted mainly of mitochondria and nucleus fragments. The supernatant was again centrifuged at 45,000 rpm for 80 min at 4 °C using a Beckman TM Optima TM L90K ultracentrifuge on a 45Ti rotor. The proteins in this supernatant, consisting of soluble cytosolic proteins, were precipitated overnight with ice-cold acetone at -20 °C. After centrifugation for 10 min at 7,500 rpm (4 °C) (Beckman TM Allegra 64R centrifuge on a F1010 rotor), the pellet of cytosolic proteins was dried under vacuum (SpeedVac, Thermo Fischer Scientific) and stored at -20 °C until further analysis.

The cytosolic proteins were then solubilized in labeling buffer (7 M urea, 2 M thiourea, 30 mM Tris, 2 % w/v 3-[(3-cholamidopropyl)dimethylammonio]-1-propanesulfonate (CHAPS), 2 % w/v 3-[N,N-Dimethyl(3-myristoylaminopropyl)ammonio]propanesulfonate (ASB14). After adjusting the pH of the sample solutions [pH 8.5 - 9], the protein concentrations were determined using the 2D Quant kit (GE Healthcare, UK) with bovine serum albumin (2 mg/mL) as standard.

### 2D-DIGE, imaging of gels and protein identification

Cytosolic proteins were labeled with the CyDyes minimal labeling method (GE Healthcare, UK). 2D-DIGE separation, imaging of the gels, picking and identification of the spot of interest were carried out as described before with minor modifications [75, 76]. The labeling was performed according to the manufacturer’s instructions. Forty μg of cytosolic proteins were used for each sample and labeled using the CyDyes minimal labeling method (GE Healthcare, UK). The internal standard was constituted by an equal fraction of each sample included in the experiment in order to correct the quantification of the proteins for potential uneven loading and electrophoretic conditions. Briefly, each sample plus internal standard were individually labeled for 30 min on ice in the dark with 320 pmol of either Cy3, Cy5 (for the analytical samples) or Cy2 (for the internal standard). Labeling reaction was stopped by the addition of 1 μL 10 mM lysine solution and incubation for 15 min on ice in the dark. Labeled samples were pooled such that each pool contained an equal ratio of proteins marked with Cy2, Cy3, and Cy5: two samples of 40 μg each (one labeled with Cy3 and one labeled with Cy5) and 40 μg of internal standard were then mixed and their final volume was adjusted to 120 μL using lysis buffer (7 M urea, 2 M thiourea, 2 % w/v CHAPS, 2 % w/v ASB14). Prior to electrophoresis, ampholytes (Biolytes 3-10, 3 % v/v, BioRad, Belgium) and Destreak reagent (0.6 % v/v, GE Healthcare, Belgium) were added to each tube.

Each pool was then loaded onto a strip for the isoelectric separation (1^st^ dimension). After re-hydration of the strips with 450 μL of Destreak re-hydration solution (GE Healthcare) for at least 12 h, the samples were directly applied on re-hydrated strips via sample loading cups. Separation was achieved using an Ettan IPGphor III (GE Healthcare) at 20 °C allowing the strip to reach a total electric current of 80 k Vh in 25 h. Following the 1^st^ dimension, proteins were reduced and alkylated by incubating the strip in two consecutive steps of 15 min at room temperature in an equilibration buffer (2DGel DALT, SERVA electrophoresis gmbh, Germany) containing 1 % DTT (Sigma, Belgium) and 2.5 % iodoacetamide (Sigma, Belgium), respectively. Equilibrated strips were orthogonally loaded on large format precast gels (24 cm 2D DALT NF large gel 12.5 %, SERVA electrophoresis gmbh, Germany) using the Flap cassette system (sealed with agarose) and subjected to electrophoretic separation with an Ettan DALT XII system (GE Healthcare, Belgium) by applying 0.5 W/gel for 2 h and then then 2.5 W/gel for 14 h at 25 °C (2^nd^ dimension). Acquisition of gel images was carried out using a Typhoon 9400 (GE Healthcare) at a special resolution of 100 μm. CyDyes were visualized using excitation at 488 nm, 532 nm, and 633 nm (Cy2, Cy3 and Cy5, respectively) and emission at 520 nm, 610 nm and 670 nm (Cy2, Cy3 and Cy5, respectively). Gels were analyzed for the differentially abundant proteins by the DeCyder 2D Differential Analysis v.7.0 software package (GE Healthcare). Criteria for the selection were: spot present in at least 75 % of the spot maps, fold change of at least ±1.3, statistical significance (*P* ≤ 0.05).

### Protein identification by MALDI-TOF/TOF

Spots of interest were used to generate a “pick list” which was submitted to the Ettan Spot Handling Workstation (GE Healthcare) for automatic picking, trypsin digestion and spotting of the peptides on the MALDI target plates with an equal volume of α-cyano-4-hydroxy cinnamic acid (HCCA). On spots of interest a combined approach of protein mass fingerprint (PMF) and MS/MS using the Applied Biosystems MALDI-TOF/TOF 4800 Proteomics Analyser was applied. Calibration was carried out with the peptide mass calibration kit 4700 (Applied Biosystems, Belgium). For each spot PMF spectra were acquired and up to 8 MS/MS fragmentations were allowed on the most abundant precursors. Protein identification was achieved by searching the acquired spectra against the NCBInr database (version 20100924with 11888344 sequences; 4060865000 residues) with “*Homo sapiens*” as taxonomy (541459 sequences, downloaded on May 2011), using GPS Explorer Software v3.6 (Applied Biosystems) including MASCOT (Matrix Science, www.matrixscience.com, London, UK). Settings chosen for the dataset search were: 150 ppm tolerance on PMF, 0.75 Da tolerance for parent ion, up to two missed cleavages allowed, carboxyamidomethylation of cysteine as fixed modification, oxidation of methionine and oxidation of tryptophan (single oxidation, double oxidation and kynurenin) as variable modification. Proteins with probability-based MOWSE scores (*P* < 0.05) were considered to be positively identified.

Univariate statistical analysis and multivariate analysis, including principal component analysis (PCA) of differentially abundant proteins and KEGG enrichment analysis, were carried out using the EDA module which is present inside the Decyder 7.0 software package.

### Statistical analysis

The data are expressed as mean values with standard deviations of three independent experiments each containing 3 replicates. Data were analyzed with SigmaPlot 12 (Systat Software, Inc. SigmaPlot for Windows) and SPSS (IBM SPSS Statistics for Windows, Version 21.0. Armonk, NY: IBM Corp) using a general linear model (univariate analysis) with a Tukey’s post Hoc test for comparison of means. When necessary the data was transformed to achieve normal distribution and equal variances. Differences among means were considered to be significant at *P* < 0.05.

## Additional file

Additional file 1: Figure S1. Mucus layer characterization (Alcian blue staining). **Figure S2.** Mucus layer characterization (Toluidine blue staining and TEM). **Figure S3.** Mucus layer characterization (Toluidine blue staining, top view). **Figure S4.** Cell monolayer integrity evaluation (TEER). **Figure S5.** Cell-free DCFH-DA assay. **Figure S6.** TEM images of cells in co-culture exposed to Ag particles. **Figure S7.** Hierarchical clustering. **Table S1.** Detailed information on protein identification. **Table S2.** Cellular Ag content determination. **Table S3.** KEGG enrichment analysis. (DOCX 1.17 mb)

## Abbreviations

Ag NPs: Silver nanoparticles
DCFH-DA: Dichlorofluorescin diacetate
2D-DIGE: Two-dimensional difference in gel electrophoresis
DLS: Dynamic light scattering
FBS: Fetal bovine serum
ICP-MS: Inductively coupled plasma mass spectrometry
IL-8: Interleukine-8
ROS: Reactive oxygen species
MALDI-TOF/TOF: Matrix assisted laser desorption ionization - Time of flight/ Time of flight
NanoSIMS: Secondary ion mass spectrometry
TEER: Transepithelial electrical resistance
TEM: Transmission electron microscopy
