# Supplementary material for: Effects of silver nanoparticles and ions on a co-culture model for the gastrointestinal epithelium
Source: Part Fibre Toxicol. 2016 Feb 17;13:9. doi: 10.1186/s12989-016-0117-9 (PMC4756536; doi:10.1186/s12989-016-0117-9)
Supplement: Additional file 1: Figure S1. — Mucus layer characterization (Alcian blue staining). Figure S2. Mucus layer characterization (Toluidine blue staining and TEM). Figure S3. Mucus layer characterization (Toluidine blue staining, top view). Figure S4. Cell monolayer integrity evaluation (TEER). Figure S5. Cell-free DCFH-DA assay. Figure S6. TEM images of cells in co-culture exposed to Ag particles. Figure S7. Hierarchical clustering. Table S1. Detailed information on protein identification. Table S2. Cellular Ag content determination. Table S3. KEGG enrichment analysis. (DOCX 1.17 mb) [file 12989_2016_117_MOESM1_ESM.docx]

**Additional Files**

**Effects of silver nanoparticles and ions on a co-culture model for the gastrointestinal epithelium**

Anastasia Georgantzopoulou^1,8^, Tommaso Serchi^1^, Sébastien Cambier^1^, Céline C. Leclercq^1^, Jenny Renaut^1^, Jia Shao^1,2^, Marcin Kruszewski^3,4^, Esther Lentzen^5^, Patrick Grysan^5^, Santhana Eswara^5^, Jean-Nicolas Audinot^5^, Servane Contal^1^, Johanna Ziebel^1^, Cédric Guignard^1^, Lucien Hoffmann^1^, AlberTinka J. Murk^6,7^, Arno C. Gutleb^1,*^

^1^Environmental Research and Innovation (ERIN) Department, Luxembourg Institute of Science and Technology (LIST), 5 avenue des Hauts-Forneaux, L-4362 Esch-sur-Alzette Luxembourg

^2^RIKILT- Institute of Food Safety, Wageningen UR, P.O. Box 230, NL-6700 AE Wageningen, the Netherlands

^3^Faculty of Medicine, University of Information Technology and Management in Rzeszow, Sucharskiego 2, 35-225 Rzeszow, Poland

^4^Institute of Nuclear Chemistry and Technology, Centre for Radiobiology and Biological Dosimety, Dorodna 16, 03-195 Warszawa, Poland

^5^ Materials Research and Technology Department (MRT), Luxembourg Institute of Science and Technology (LIST), 5 avenue des Hauts-Forneaux, L-4362 Esch-sur-Alzette Luxembourg

^6^Sub-department of Environmental Technology, Wageningen, The Netherlands

^7^IMARES Wageningen UR Institute for Marine Resources & Ecosystem Studies, P.O. Box 57, NL 1780 AB Den Helder, The Netherlands

^8^Current address: Norwegian Institute for Water Research (NIVA), Gaustadalléen 21, NO-0349 Oslo, Norway

* Corresponding Author: Arno C. Gutleb, Environmental Research and Innovation (ERIN) Department, Luxembourg Institute of Science and Technology (LIST), 5 avenue des Hauts-Forneaux, L-4362 Esch-sur-Alzette Luxembourg. Tel: (+352) 470261481, Fax: (+352) 470264, E-mail: [arno.gutleb@list.lu](mailto:arno.gutleb@list.lu)

**Materials and Methods**

# Co-culture System Characterization- Alcian Blue Staining (mucus localization): Caco-2/TC7:HT29-MTX cells in co-culture at increasing ratios (100:0, 90:10, 50:50 and 0:100) were seeded in 6-well plates at an initial total cell concentration of 1.2x10^5^ cells/mL and cultured for 14 days as required for cell differentiation and formation of tight junctions. The culture medium was removed after the 14-day period, the monolayers were washed once with PBS and fixed with 6% formaldehyde in 0.27 M CaCl_2_, pH 4.0 at 4 °C for 1 hour as previously described [1]. Cell monolayers were washed with PBS and then stained with Alcian blue (pH 2.5), that stains the acid muco-substances attached to HT29-MTX cells blue. Cells were observed under a microscope and pictures of the stained cells were acquired.

*Co-culture System Characterization- Toluidine Blue Staining- (mucus coverage)*: Caco-2/TC7:HT29-MTX cells in co-culture at increasing ratios were seeded onto polycarbonate 0.4 µM pore size trans-well inserts at an initial concentration of 1.2x10^5^ cells/mL and cultured for 14 days. The membranes were cut into strips and stained with 0.25% toluidine blue in PBS for 5 minutes and placed transversally in multiwell chambered coverslips (Sigma-Aldrich) for observation under a microscope (the cell monolayer is stained blue whereas the mucus layer purple) as previously described [2].

*Cell Monolayer Integrity Evaluation: Transepithelial Electrical Resistance (TEER)*

Caco-2/TC7, HT29-MTX cells and cells in co-culture at a ratio of 90:10 (Caco-2/TC7:HT29:MTX) were seeded onto polycarbonate 0.4 µM pore size 12-well hanging trans-well inserts at an initial concentration of 1.2x10^5^ cells/mL (0.5 mL/insert) and cultured for 14 days. The medium was changed every other day in the apical (0.5 mL) and the basolateral (1.5 mL) compartment. The integrity of the monitored using an epithelial volt-ohmmeter fitted with planar electrodes (Millipore, MILLICELL®-ERS) during the medium change. At the end of the growth period, fresh growth medium containing the Ag NPs 20 nm, 200 nm or AgNO_3_ was added to the apical compartment of each insert. After a 24-hour exposure to the TEER of the monolayers were measured.

*TEM investigation of the co-culture system exposed to Ag NPs:* All the steps described below were directly performed in the trans-well insert where the cells were seeded to obtain a transversal picture of the fully differentiated system. The co-culture of Caco-2/TC7 and HT29-MTX at a 90:10 ratio was seeded in 12-well plates and after 14 days in culture the cells were exposed to 30 mg/L Ag 20 and 200 nm particles for 24 hours. The cells were washed with PBS and then fixed with 5% glutaraldehyde in PBS overnight at 4°C. The glutaraldehyde was removed and the cells were washed with PBS. They were then post fixed with 1% OsO_4_ in milliQ water for 2 hours. After an additional washing step with PBS, the cells were dehydrated with increasing ethanol concentrations (30%, 50%, 70%, 90% and 100% ethanol two times). They were finally embedded in epoxy resin (SPURR) in molds (easy molds, Ted Pella, Inc). The samples were cut to 80 nm thin sections (Leica Ultracut UCT, Le Pecq Cedex, France) and deposited on copper TEM grids with Formvar/Carbon support films for TEM analysis.

The TEM used in this investigation was a FEI Tecnai F20 with TWIN objective lens. The acceleration voltage was set to 120 kV. The images were acquired in the bright-field mode using a Gatan Ultrascan CCD camera.


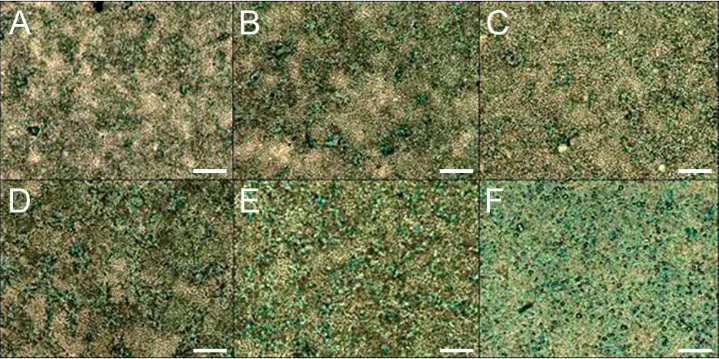


**Figure S1** Mucus layer localization. Mucus layer stained with alcian blue in Caco-2/TC7:HT29-MTX mixed cultures 14 days after seeding with increasing densities of HT29-MTX cells (A) 0%, (B) 10%, (C) 25%, (D) 50%, (E) 75% and (F) 100%. Scale bar is 50 µm.


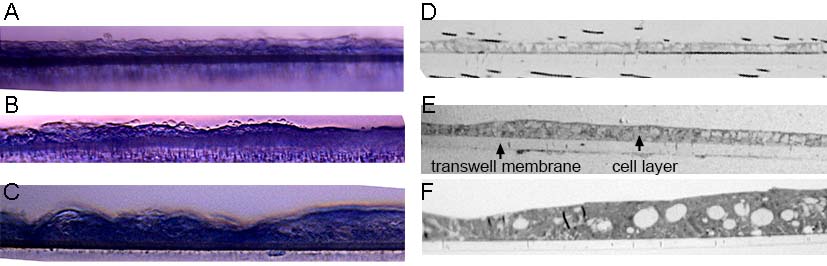


**Figure S2** Profile of the Caco-2/TC7:HT29-MTX cells after 14 days in culture. The HT29-MTX cells were seeded at increasing densities (A, D) 0%, (B, E) 10%, (C, F) 100%. Toluidine blue staining was performed (A, B, C, 20X magnification) and TEM images of the cells grown in the inserts were taken (D, E, F).


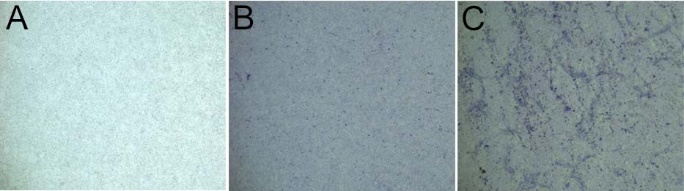


**Figure S3** Top view of the Toluidine blue stained cells grown in transwell inserts after 14 days in culture. The HT29-MTX cells were seeded at increasing densities (A) 0%, (B) 10%, (C) 100% (5X magnification).


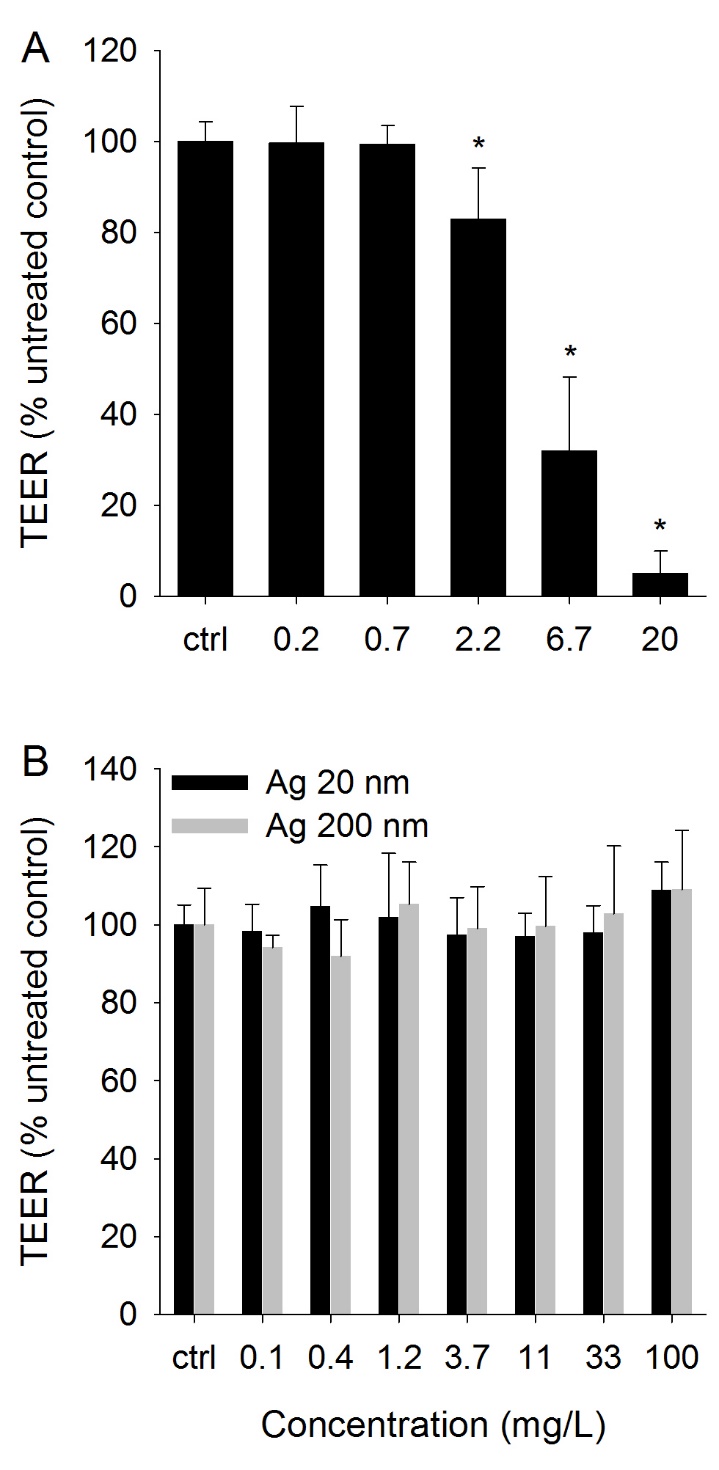


**Figure S4** Effects on trans-epithelial electrical resistance (TEER). Effects of (A) AgNO_3_ and (B) Ag NPs 20 and 200 nm on the trans-epithelial electrical resistance (TEER) expressed as percentage of untreated control. Differentiated cells (after 14 days of culture) were exposed for 24 hours. Error bars represent the mean ± SD of 2 independent experiments performed in triplicate. Significant differences from respective untreated controls are marked with asterisks (* for P< 0.05).


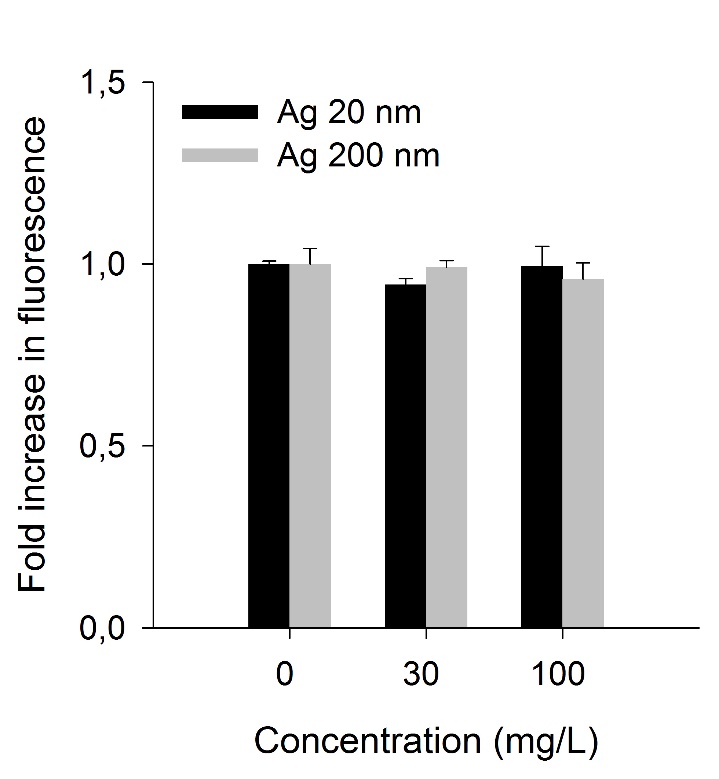


**Figure S5** Effects of Ag 20 and 200 nm particles on the DCF production in a cell free DCFH-DA assay. Results are expressed as fold increase in DCF fluorescence over the control (DCFH-DA in medium). Error bars represent the mean ± SD of triplicates.


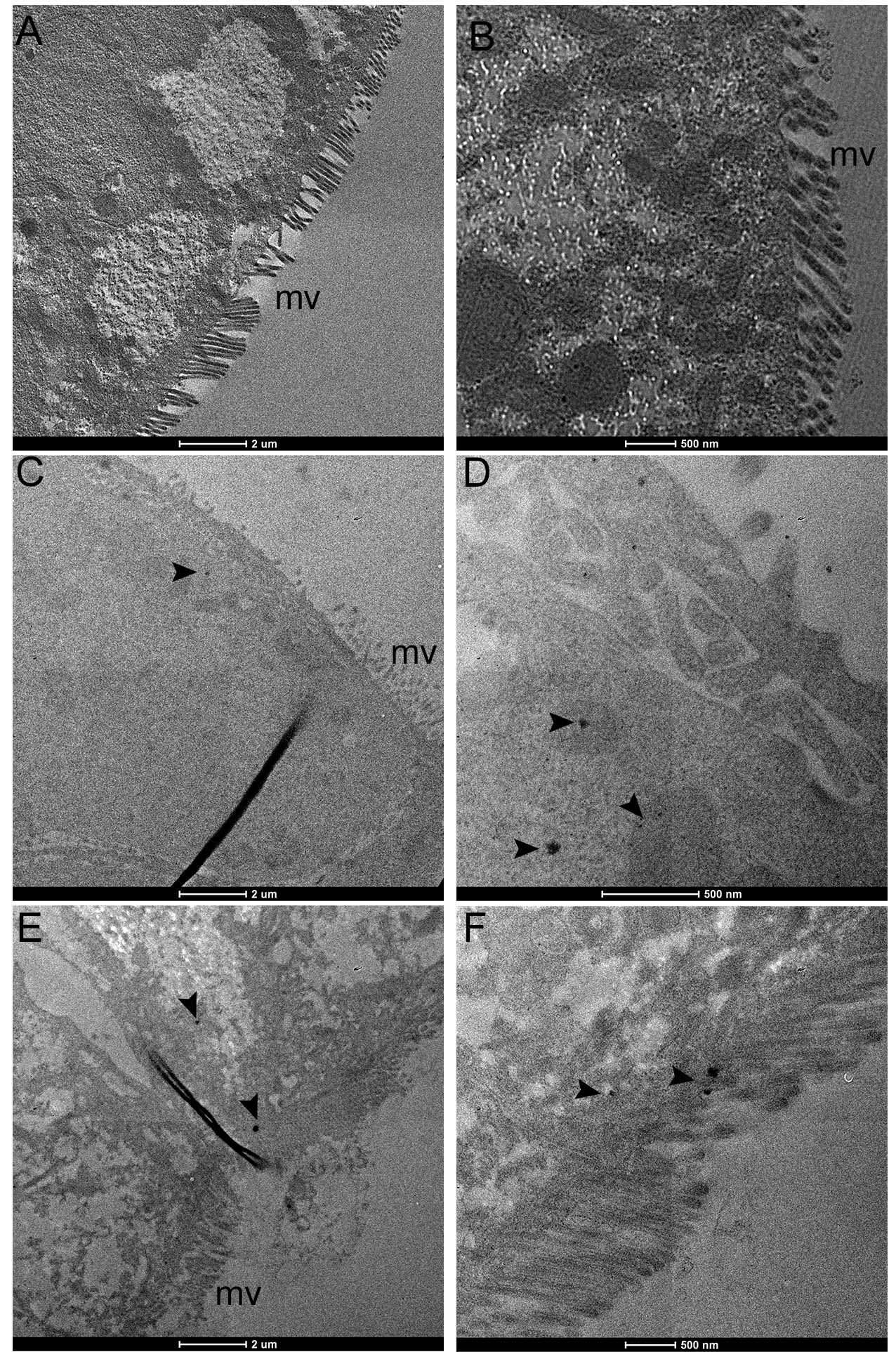


**Figure S6** TEM images of Caco-2/TC7:HT29-MTX cells in 90:10 co-culture. The cells in co-culture were exposed to (C-D) Ag 20 nm and (E-F) Ag 200 nm for 24 hours while (A-B) represents the untreated control cells. Mv: microvilli. Scale bars are 2 μm (A, C, E) and 500 nm (B, D, F).


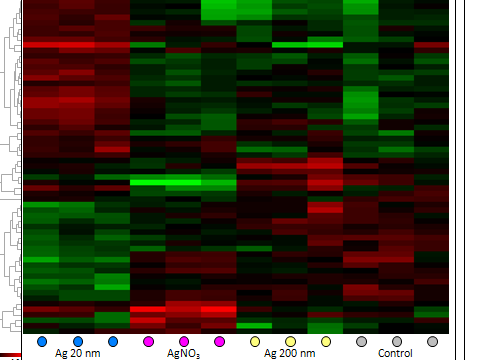


**Figure S7** Hierarchical clustering of differentially expressed proteins. Hierarchical clustering of differentially expressed proteins in Caco-2/TC7:HT29-MTX cells in 90:10 co-culture exposed to 1 mg/L of Ag NPs 20 nm, 200 nm or AgNO_3_ for 24 hours.

**Table S1** Identification table. For each identified protein UniProt Accession number, UniProt ID, NCBI accession number, percentage of sequence coverage, cumulative MOWSE score with corresponding p-value, number of queries matched, theoretical molecular mass and isoelectric points are reported (Table S1.xls).

**Table S2** Cellular Ag content (ICP-MS). The Caco-2/TC7, HT29-MTX and Caco-2/TC7:HT29-MTX cells in 90:10 co-culture were exposed to 100 mg/L of Ag NPs 20 nm, 200 nm or 20 mg/L AgNO_3_ for 24 hours. The cells in co-culture were also treated with 1 mg/L Ag NPs or AgNO_3_ for comparison. Results are shown as average ± SD of triplicates.

| Cell line | Exposure | µg Ag/sample |
| --- | --- | --- |
| Caco-2/TC7 | Ctrl | <0.00075 |
|  | AgNO_3_ (20 mg/L) | 0.02 ± 0.02 |
|  | Ag 20 (100 mg/L) | 0.45 ± 0.11 |
|  | Ag 200 (100 mg/L) | 0.78 ± 0.21 |
| HT29-MTX | Ctrl | <0.00075 |
|  | AgNO_3_ (20 mg/L) | 0.26 ± 0.05 |
|  | Ag 20 (100 mg/L) | 0.45 ± 0.09 |
|  | Ag 200 (100 mg/L) | 0.70 ± 0.42 |
| 90:10 co-culture | Ctrl | <0.00075 |
|  | AgNO_3_ (1 mg/L) | 0.16 ± 0.03 |
|  | AgNO_3_ (20 mg/L) | 0.07 ± 0.04 |
|  | Ag 20 (1 mg/L) | 0.13 ± 0.03 |
|  | Ag 20 (100 mg/L) | 0.44 ± 0.31 |
|  | Ag 200 (1 mg/L) | 0.33 ± 0.06 |
|  | Ag 200 (100 mg/L) | 0.53 ± 0.39 |

**References**

1. Mahler GJ, Shuler ML, Glahn RP: **Characterization of Caco-2 and HT29-MTX cocultures in an *in vitro* digestion/cell culture model used to predict iron bioavailability.** *J Nutr Biochem* 2009, **20**:494–502.

2. Wikman A, Karlsson J, Carlstedt I, Artursson P: **A drug absorption model based on the mucus layer producing human intestinal goblet cell line HT29-H.** *Pharm Res* 1993, **10**:843–52.
